# Single-inhaler fluticasone furoate/umeclidinium/vilanterol (FF/UMEC/VI) triple therapy versus tiotropium monotherapy in patients with COPD

**DOI:** 10.1038/s41533-021-00241-z

**Published:** 2021-05-25

**Authors:** Sandeep Bansal, Martin Anderson, Antonio Anzueto, Nicola Brown, Chris Compton, Thomas C. Corbridge, David Erb, Catherine Harvey, Morrys C. Kaisermann, Mitchell Kaye, David A. Lipson, Neil Martin, Chang-Qing Zhu, Alberto Papi

**Affiliations:** 1 The Lung Center, Penn Highlands Healthcare, Du Bois, PA USA; 2 Karolinska Institutet, Stockholm, Sweden; 3 Division of Pulmonary Diseases and Critical Care Medicine, School of Medicine, The University of Texas Health Science Center, San Antonio, TX USA; 4 Audie L. Murphy Memorial VA Hospital, South Texas Veterans Health Care System, San Antonio, TX USA; 5 GSK, Stockley Park West, Iron Bridge Road North, West Drayton, Uxbridge, Middlesex UK; 6 GSK, Brentford, Middlesex UK; 7 GSK, Research Triangle Park, NC USA; 8 Feinberg School of Medicine, Northwestern University, Chicago, IL USA; 9 VitaLink Research Gaffney, Gaffney, SC USA; 10 GSK, Collegeville, PA USA; 11 Minnesota Lung Center, Minneapolis, MN USA; 12 Perelman School of Medicine, University of Pennsylvania, Philadelphia, PA USA; 13 University of Leicester, Leicester, Leicestershire UK; 14 Respiratory Unit, Department of Morphology, Surgery and Experimental Medicine, University of Ferrara, Ferrara, FE Italy

**Keywords:** Chronic obstructive pulmonary disease, Therapeutics, Medical research

## Abstract

Chronic obstructive pulmonary disease (COPD) treatment guidelines do not currently include recommendations for escalation directly from monotherapy to triple therapy. This 12-week, double-blind, double-dummy study randomized 800 symptomatic moderate-to-very-severe COPD patients receiving tiotropium (TIO) for ≥3 months to once-daily fluticasone furoate/umeclidinium/vilanterol (FF/UMEC/VI) 100/62.5/25 mcg via ELLIPTA (*n* = 400) or TIO 18 mcg via HandiHaler (*n* = 400) plus matched placebo. Study endpoints included change from baseline in trough forced expiratory volume in 1 s (FEV_1_) at Days 85 (primary), 28 and 84 (secondary), health status (St George’s Respiratory Questionnaire [SGRQ] and COPD Assessment Test [CAT]) and safety. FF/UMEC/VI significantly improved trough FEV_1_ at all timepoints (Day 85 treatment difference [95% CI] 95 mL [62–128]; *P* < 0.001), and significantly improved SGRQ and CAT versus TIO. Treatment safety profiles were similar. Once-daily single-inhaler FF/UMEC/VI significantly improved lung function and health status versus once-daily TIO in symptomatic moderate-to-very-severe COPD patients, with a similar safety profile.

## Introduction

Chronic obstructive pulmonary disease (COPD) is a major cause of chronic morbidity and mortality worldwide^1^. It is a preventable and treatable disease, characterized by persistent respiratory symptoms and airflow limitation^1^. Determining the appropriate treatment requires a thorough understanding of the disease at an individual level, and assessments should cover symptomatology, exacerbation risk, and the degree of airflow limitation^1^. Treatment should then be tailored based on these disease characteristics and escalated, as needed, should the patient experience clinically significant symptoms and/or exacerbations^1^.

The Global Initiative for Chronic Obstructive Lung Disease (GOLD) 2020 strategy document recommends escalating from monotherapy (long-acting muscarinic antagonist [LAMA] or long-acting β_2_-agonist [LABA]) to dual therapy (LAMA/LABA or inhaled corticosteroid [ICS]/LABA) or from dual therapy to triple therapy (ICS/LAMA/LABA) for patients who continue to experience clinically significant symptoms and/or exacerbations on their current maintenance therapy^1^. In real-life management of COPD, patients are often escalated to triple therapy by adding ICS/LABA to LAMA monotherapy, with one study showing that over a quarter of patients with newly diagnosed COPD progress to triple therapy within 24 months of diagnosis^2,3^. However, despite its occurrence in clinical practice, recommendations for escalation from monotherapy directly to triple therapy are currently not included in treatment guidelines. The reasons for this are varied but include heterogeneous endpoints in the clinical studies performed to date and a lack of updated recommendations based on the current body of evidence.

In a number of clinical studies, stepping up from LAMA monotherapy to ICS/LAMA/LABA triple therapy improved lung function compared with LAMA monotherapy in patients with symptomatic COPD^4–10^. Triple therapy versus LAMA monotherapy also led to statistically significant decreases (improvements) in St George’s Respiratory Questionnaire (SGRQ) score^5–9^, which measures health-related quality of life^11^, while a study with moderate/severe exacerbation rate as the primary endpoint showed that single-inhaler triple therapy led to a significant reduction in moderate/severe exacerbation rate versus LAMA monotherapy^5^. In most of these studies, triple therapy was administered using multiple inhalers, therefore evidence comparing single-inhaler triple therapy to LAMA monotherapy would be of clinical relevance. It has previously been demonstrated in Phase III trials that single-inhaler triple therapy with fluticasone furoate/umeclidinium/vilanterol (FF/UMEC/VI) significantly reduces moderate/severe exacerbations and improves lung function and health status compared with dual therapy with FF/VI or UMEC/VI (IMPACT trial) or budesonide/formoterol (FULFIL trial) in patients with symptomatic COPD who are at risk of exacerbations, while the safety profile of triple therapy reflected the known profiles of its components^12,13^. The current Phase IV study (study 207626) evaluated the efficacy and safety of once-daily single-inhaler FF/UMEC/VI therapy versus once-daily LAMA monotherapy with tiotropium (TIO) in patients with symptomatic COPD with moderate-to-very-severe airflow limitation.

## Results

### Trial population

**Fig. 1:**
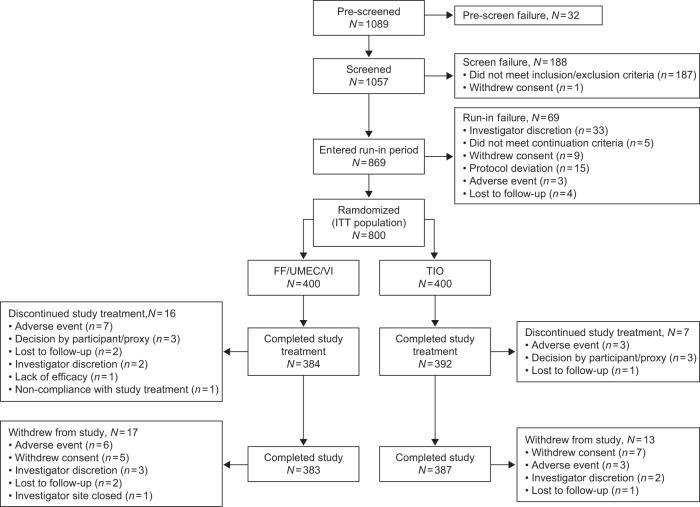
Study design. FF fluticasone furoate, ITT intent-to-treat, TIO tiotropium, UMEC umeclidinium, VI vilanterol.

The ITT population included 800 patients who underwent randomization (FF/UMEC/VI, *N* = 400; TIO, *N* = 400; Fig. 1). Nearly all patients (96%) completed all protocol-defined study visits, with similar discontinuation and withdrawal rates between treatment groups (Fig. 1). Baseline characteristics and demographics were similar between the two treatment groups (Table 1).

**Table 1:** Patient demographics and baseline characteristics (ITT population).

	FF/UMEC/VI *N* = 400	TIO *N* = 400	Total *N* = 800
Age, years, mean (SD)	66.2 (8.08)	66.1 (7.78)	66.2 (7.93)
Male, *n* (%)	274 (69)	269 (67)	543 (68)
BMI, kg/m^2^, mean (SD)	27.5 (6.1)	27.2 (5.3)	27.4 (5.7)
Current smoker at screening, *n* (%)	189 (47)	192 (48)	381 (48)
Lung function at screening, mean (SD)			
Post-bronchodilator FEV_1_, mL^a^	1434 (493)	1443 (504)	1439 (498)
Post-bronchodilator percent predicted FEV_1_, %^a^	49.8 (14.0)	50.2 (14.2)	50.0 (14.1)
Post-bronchodilator FEV_1_/FVC ratio^a^	0.493 (0.109)	0.502 (0.106)	0.498 (0.107)
Percent reversibility to salbutamol, %^b^	8.7 (13.4)	8.6 (11.6)	8.6 (12.5)
COPD exacerbations in the previous 12 months, *n* (%)			
Moderate COPD exacerbations			
0	145 (36)	151 (38)	296 (37)
1	44 (11)	40 (10)	84 (11)
≥2	211 (53)	209 (52)	420 (53)
Severe COPD exacerbations			
0	318 (80)	312 (78)	630 (79)
1	72 (18)	77 (19)	149 (19)
≥2	10 (3)	11 (3)	21 (3)
CAT score at screening, mean (SD)^c^	20.7 (5.32)	20.5 (5.16)	20.6 (5.24)
GOLD grade, *n* (%)^a^			
Grade 1 (mild)	1 (<1)	0	1 (<1)
Grade 2 (moderate)	184 (46)	195 (49)	379 (48)
Grade 3 (severe)	180 (45)	173 (43)	353 (44)
Grade 4 (very severe)	32 (8)	30 (8)	62 (8)

*BMI* body mass index, *CAT* COPD Assessment Test, *COPD* chronic obstructive pulmonary disease, *FEV*_*1*_ forced expiratory volume in 1 s, *FF* fluticasone furoate, *FVC* forced vital capacity, *GOLD* Global Initiative for Chronic Obstructive Lung Disease, *ITT* intent-to-treat, *SD* standard deviation, *TIO* tiotropium, *UMEC* umeclidinium, *VI* vilanterol.

^a^FF/UMEC/VI: *n* = 397, TIO: *n* = 398, total: *n* = 795.

^b^FF/UMEC/VI: *n* = 392, TIO: *n* = 391, total: *n* = 783.

^c^TIO: *n* = 399, total: *n* = 799.

### Efficacy

**Fig. 2:**
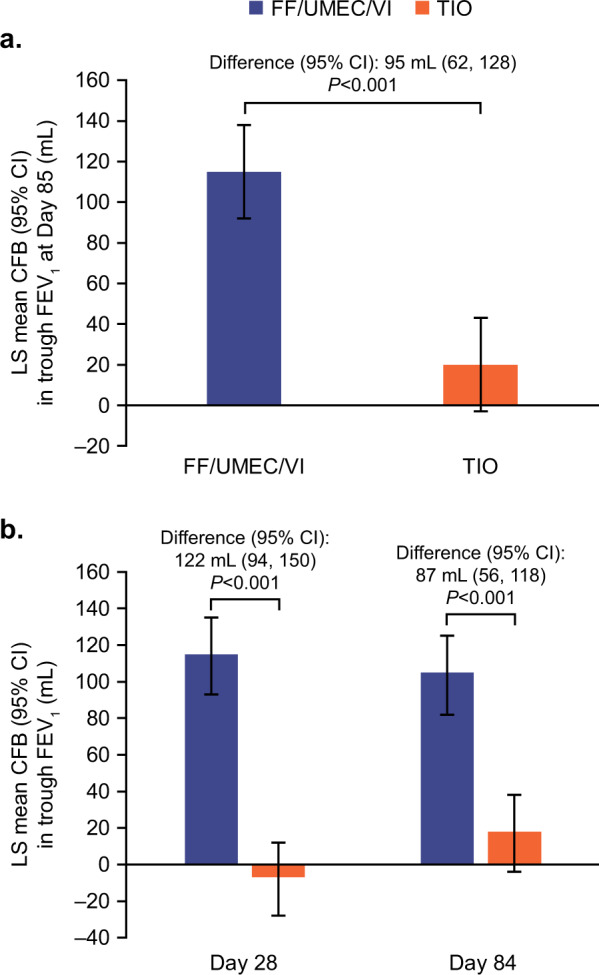
Trough FEV_1_ (ITT population). Least squares mean (95% CI) change from baseline in trough FEV_1_ at **a** Day 85 and **b** Days 28 and 84. CFB change from baseline, CI confidence interval, FEV_1_ forced expiratory volume in 1 s, FF fluticasone furoate, ITT intent-to-treat, LS least squares, TIO tiotropium, UMEC umeclidinium, VI vilanterol.

The mean change from baseline in trough FEV_1_ at Day 85 was significantly greater with FF/UMEC/VI versus TIO, with a treatment difference of 95 mL (95% confidence interval [CI]: 62, 128; *P* < 0.001; Fig. 2a).

The mean change from baseline in trough FEV_1_ was significantly greater with FF/UMEC/VI versus TIO at both Day 28 and Day 84, with treatment differences (95% CI) of 122 mL (94, 150; *P* < 0.001) and 87 mL (56, 118; *P* < 0.001), respectively (Fig. 2b).

**Fig. 3:**
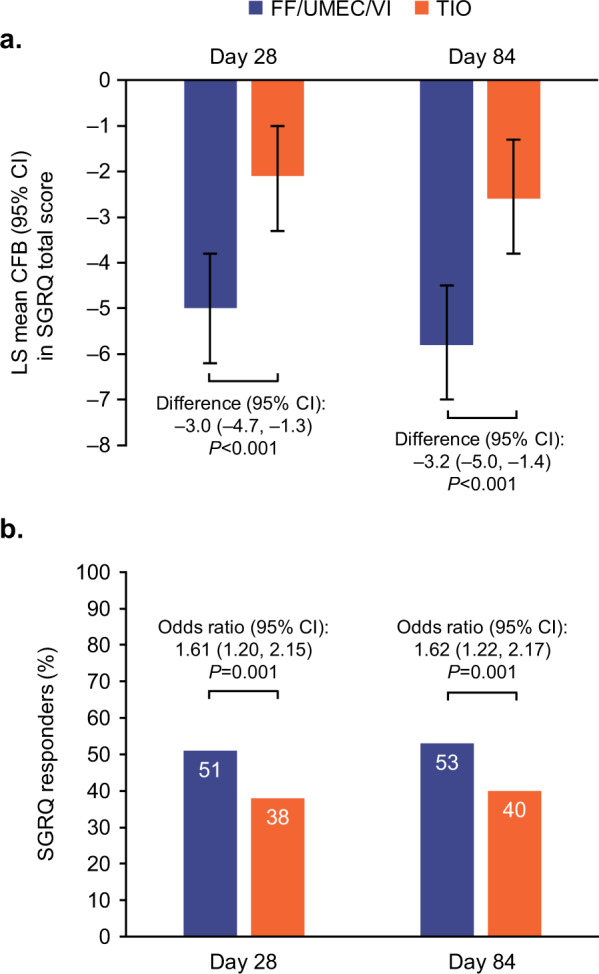
SGRQ total score (ITT population). **a** Least squares mean (95% CI) change from baseline in SGRQ total score and **b** proportion of SGRQ responders (≥4-point decrease in SGRQ total score) at Day 28 and Day 84. CFB change from baseline, CI confidence interval, FF fluticasone furoate, ITT intent-to-treat, LS least squares, SGRQ St George’s Respiratory Questionnaire, TIO tiotropium, UMEC umeclidinium, VI vilanterol.

A significantly greater mean decrease from baseline in SGRQ total score was observed with FF/UMEC/VI versus TIO at both Day 28 and Day 84. The between treatment differences (95% CI) were −3.0 (−4.7, −1.3; *P* < 0.001) and −3.2 (−5.0, −1.4; *P* < 0.001), respectively (Fig. 3a). The odds of being a SGRQ total score responder were significantly greater with FF/UMEC/VI versus TIO at Day 28 (odds ratio [OR] [95% CI]: 1.61 [1.20, 2.15]; *P* = 0.001) and Day 84 (OR [95% CI]: 1.62 [1.22, 2.17]; *P* = 0.001; Fig. 3b).

**Fig. 4:**
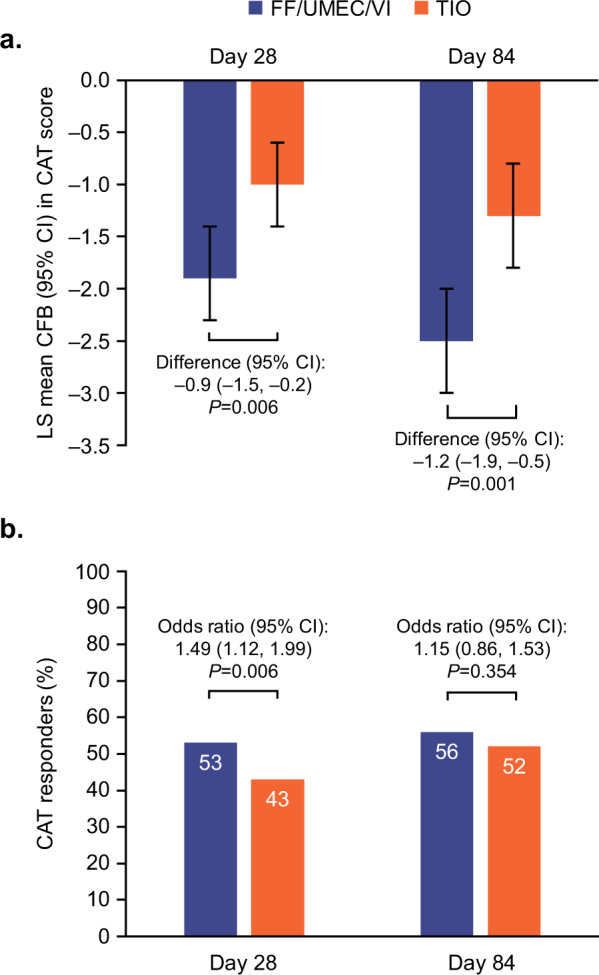
CAT Score (ITT population). **a** Least squares mean (95% CI) change from baseline in CAT score and **b** proportion of CAT responders (≥2-point decrease in CAT score) at Day 28 and Day 84. CAT COPD Assessment Test, CFB change from baseline, CI confidence interval, FF fluticasone furoate, ITT intent-to-treat, LS least squares, TIO tiotropium, UMEC umeclidinium, VI vilanterol.

CAT score decreased significantly from baseline with FF/UMEC/VI versus TIO at Days 28 and 84. Between treatment differences (95% CI) were −0.9 (−1.5, −0.2; *P* = 0.006) and −1.2 (−1.9, −0.5; *P* = 0.001), respectively (Fig. 4a). For CAT responder analyses, ORs were in favor of FF/UMEC/VI at both Day 28 and 84. Statistical significance in favor of FF/UMEC/VI was achieved at Day 28 (OR [95% CI]: 1.49 [1.12, 1.99]; *P* = 0.006) but not Day 84 (OR [95% CI]: 1.15 [0.86, 1.53]; *P* = 0.354; Fig. 4b).

In total, 27 (7%) and 43 (11%) patients receiving FF/UMEC/VI and TIO, respectively, experienced a moderate/severe exacerbation during the 12-week study period. Severe exacerbations were seen in 5 (1%) and 3 (<1%) patients receiving FF/UMEC/VI and TIO, respectively.

The FEV_1_ < 50% predicted subgroup comprised 212 patients receiving FF/UMEC/VI and 203 patients receiving TIO; the FEV_1_ ≥ 50% predicted subgroup comprised 185 patients receiving FF/UMEC/VI and 195 patients receiving TIO (Table 2). Demographics at screening were similar across FEV_1_ subgroups, although lung function parameters differed substantially. Patients in the ≥50% subgroup experienced substantially more moderate COPD exacerbations in the 12 months prior to the study (Table 2), as the study inclusion criteria required a documented history of ≥2 moderate exacerbations or 1 severe exacerbation in the last 12 months for this subgroup.

**Table 2:** Screening demographics and characteristics for FEV_1_ percent predicted subgroups (ITT population).

	Predicted FEV_1_ at screening <50% *N* = 415		Predicted FEV_1_ at screening ≥50% *N* = 380	
	FF/UMEC/VI	TIO	FF/UMEC/VI	TIO
	*n* = 212	*n* = 203	*n* = 185	*n* = 195
Age, years, mean (SD)	65.9 (8.02)	65.2 (7.44)	66.7 (8.16)	67.0 (8.05)
Male, *n* (%)	149 (70)	129 (64)	123 (66)	139 (71)
BMI, kg/m^2^, mean (SD)	27.4 (6.4)	27.0 (5.8)	27.6 (5.7)	27.4 (4.8)
Current smoker at screening, *n* (%)	104 (49)	100 (49)	82 (44)	91 (47)
Lung function at screening, mean (SD)				
Post-bronchodilator FEV_1_, mL	1137 (314)	1097 (290)	1775 (437)	1803 (420)
Post-bronchodilator percent predicted FEV_1_, %	39.2 (7.8)	38.6 (7.6)	62.1 (8.2)	62.4 (7.8)
Post-bronchodilator FEV_1_/FVC ratio	0.437 (0.099)	0.445 (0.097)	0.557 (0.081)	0.561 (0.079)
Percent reversibility to salbutamol, %^a^	10.9 (14.9)	9.8 (12.1)	6.3 (11.0)	7.4 (10.9)
COPD exacerbations in the previous 12 months, *n* (%)				
Moderate COPD exacerbations				
0	117 (55)	115 (57)	27 (15)	36 (18)
1	39 (18)	32 (16)	4 (2)	8 (4)
≥2	56 (26)	56 (28)	154 (83)	151 (77)
Severe COPD exacerbations				
0	168 (79)	163 (80)	148 (80)	147 (75)
1	41 (19)	37 (18)	31 (17)	40 (21)
≥2	3 (1)	3 (1)	6 (3)	8 (4)
CAT score at screening, mean (SD)^b^	21.6 (5.58)	21.2 (5.41)	19.7 (4.68)	19.8 (4.76)
SGRQ total score at baseline, mean (SD)^c^	53.3 (15.39)	50.0 (15.62)	46.4 (15.55)	45.5 (14.24)

*BMI* body mass index, *CAT* COPD Assessment Test, *COPD* chronic obstructive pulmonary disease, *FEV*_*1*_ forced expiratory volume in 1 s, *FF* fluticasone furoate, *FVC* forced vital capacity, *ITT* intent-to-treat, *SD* standard deviation, *SGRQ* St George’s Respiratory Questionnaire, *TIO* tiotropium, *UMEC* umeclidinium, *VI* vilanterol.

^a^Percent predicted FEV_1_ < 50%: FF/UMEC/VI, *n* = 207, TIO, *n* = 197; percent predicted FEV_1_ ≥ 50%: TIO, *n* = 194.

^b^Percent predicted FEV_1_ < 50%: TIO, *n* = 202.

^c^Percent predicted FEV_1_ < 50%: FF/UMEC/VI, *n* = 210; percent predicted FEV_1_ ≥ 50%: FF/UMEC/VI, *n* = 184, TIO, *n* = 194.

**Fig. 5:**
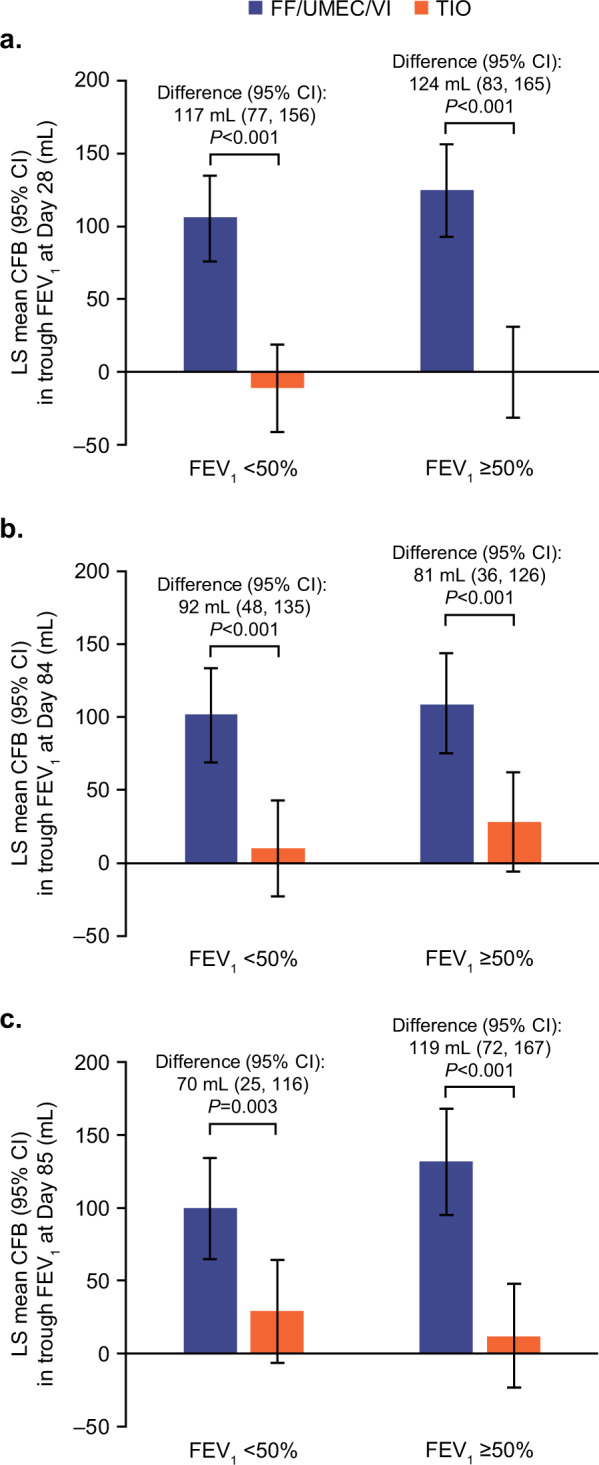
Trough FEV_1_ for percent predicted FEV_1_ at screening subgroups (ITT population). Least squares mean (95% CI) change from baseline in trough FEV_1_ at **a** Day 28, **b** Day 84, and **c** Day 85. CFB change from baseline, CI confidence interval, FEV_1_ forced expiratory volume in 1 s, FF fluticasone furoate, ITT intent-to-treat, LS least squares, TIO tiotropium, UMEC umeclidinium, VI vilanterol.

Mean change from baseline in trough FEV_1_ was significantly greater with FF/UMEC/VI versus TIO at Days 28, 84, and 85 in both subgroups (Fig. 5). Significantly greater decreases from baseline in SGRQ total score with FF/UMEC/VI versus TIO were observed at Days 28 and 84 for the FEV_1_ < 50% subgroup. For the FEV_1_ ≥ 50% subgroup, a numerical decrease in favor of FF/UMEC/VI was observed at Day 28, and the decrease with FF/UMEC/VI versus TIO was statistically significant at Day 84 (Supplementary Fig. 1). Greater decreases from baseline in CAT scores with FF/UMEC/VI versus TIO were observed at Days 28 and 84 for both subgroups, but treatment differences were statistically significant for the FEV_1_ < 50% subgroup only (Supplementary Fig. 2).

### Safety profile

The incidence of AEs, SAEs, and AESIs was similar between treatment groups, including cardiovascular effects, and there was no between-group difference in pneumonia rates (Table 3). There were no new safety findings associated with the use of an ICS, a LAMA, and a LABA in combination. Two patients died in the FF/UMEC/VI arm and one patient died in the TIO arm; these deaths were not considered to be related to study treatment.

**Table 3:** Incidence of on-treatment AEs (ITT population).

	FF/UMEC/VI		TIO	
	*N* = 400		*N* = 400	
	*n* (%)	Rate [#]	*n* (%)	Rate [#]
Total treatment exposure, patient-years	90.5		92.0	
AEs				
Any	127 (32)	2609.2 [236]	115 (29)	2695.2 [248]
Drug related	11 (3)	199.0 [18]	4 (1)	228.2 [21]
Leading to permanent discontinuation or study withdrawal^a^	7 (2)	110.6 [10]	3 (<1)	32.6 [3]
SAEs				
Any	13 (3)	187.9 [17]	10 (3)	130.4 [12]
Drug related	0	–	0	–
Leading to permanent discontinuation or study withdrawal	4 (1)	44.2 [4]	3 (<1)	32.6 [3]
Fatal	2 (<1)	22.1 [2]	1 (<1)	10.9 [1]
AESIs				
Cardiovascular effects	11 (3)	143.7 [13]	11 (3)	195.6 [18]
Decreased BMD and associated fractures	2 (<1)	22.1 [2]	0	–
LRTI excluding pneumonia	0	–	1 (<1)	10.9 [1]
Pneumonia	3 (<1)	33.2 [3]	3 (<1)	32.6 [3]

Rate is the number of events per 1000 patient-years, calculated as the number of events × 1000 divided by the total treatment exposure.

# number of events, *AE* adverse event, *AESI* adverse event of special interest, *BMD* bone mineral density, *COPD* chronic obstructive pulmonary disease, *FF* fluticasone furoate, *ITT* intent-to-treat, *LRTI* lower respiratory tract infection, *SAE* serious adverse event, *TIO* tiotropium, *UMEC* umeclidinium, *VI* vilanterol.

^a^AEs leading to permanent discontinuation or study withdrawal included pneumonia (FF/UMEC/VI *n* = 1 [<1%]; TIO *n* = 1 [<1%]), oral fungal infection (FF/UMEC/VI *n* = 1 [<1%]; TIO *n* = 0), postoperative wound infection (FF/UMEC/VI *n* = 1 [<1%]; TIO *n* = 0), hemorrhagic stroke (FF/UMEC/VI *n* = 0; TIO *n* = 1 [<1%]), ischemic stroke (FF/UMEC/VI *n* = 0; TIO *n* = 1 [<1%]), tremor (FF/UMEC/VI *n* = 1 [<1%]; TIO *n* = 0), cardiac arrest (FF/UMEC/VI *n* = 1 [<1%]; TIO *n* = 0), palpitations (FF/UMEC/VI *n* = 1 [<1%]; TIO *n* = 0), asthenia (FF/UMEC/VI *n* = 1 [<1%]; TIO *n* = 0), insomnia (FF/UMEC/VI *n* = 1 [<1%]; TIO *n* = 0), COPD (FF/UMEC/VI *n* = 1 [<1%]; TIO *n* = 0), and hyperhidrosis (FF/UMEC/VI *n* = 1 [<1%]; TIO *n* = 0).

## Discussion

This study examined the effect of single-inhaler FF/UMEC/VI triple therapy versus TIO monotherapy in patients with symptomatic COPD with moderate-to-very-severe airflow limitation, as in clinical practice patients are often escalated directly from LAMA monotherapy to triple therapy with the addition of ICS/LABA. The superiority of FF/UMEC/VI versus TIO was demonstrated for the primary endpoint of change from baseline in trough FEV_1_ at Day 85. Furthermore, significant improvements were seen in trough FEV_1_ at Days 28 and 84, with the greatest improvement at Day 28 (exceeding the MCID value of 100 mL), indicating that FF/UMEC/VI leads to early and sustained benefits in lung function in this population. These findings are in line with previous studies comparing TIO monotherapy to multiple-inhaler ICS/LAMA/LABA triple therapy in patients with COPD, all of which demonstrated statistically significant improvements in pre-dose FEV_1_ in favor of triple therapy^14^. These data are also consistent with a recent study demonstrating significant improvements in pre-dose FEV_1_ from 4 to 52 weeks following initiation of single-inhaler triple therapy (beclometasone dipropionate, formoterol fumarate, glycopyrronium bromide) compared with TIO monotherapy in patients with symptomatic COPD with FEV_1_ < 50% and a history of exacerbations^5^. However, while sustained and significant improvements with triple therapy versus LAMA monotherapy were observed in this study, the whole clinical picture and general symptom burden must be taken into account in clinical practice. For example, while this study showed greater improvements in lung function in patients with FEV_1_ ≥ 50%, these results cannot necessarily be extrapolated to improving dyspnea in patients with symptomatic COPD and a history of exacerbation but preserved lung function.

Early and sustained improvements in health status, as assessed by SGRQ total score and CAT score, were also seen in the current study, with significant decreases in both scores with FF/UMEC/VI versus TIO at Days 28 and 84. Moreover, significantly more patients achieved a ≥ 4-point decrease in SGRQ total score with FF/UMEC/VI versus TIO at both Day 28 and 84. The CAT responder analysis showed a similar trend, with an OR favoring FF/UMEC/VI at both time points, although statistical significance in favor of FF/UMEC/VI was only achieved at Day 28. This was likely due to small decreases in CAT score in the TIO group at Day 84 which tipped patients over the response threshold despite a minimal change versus Day 28, resulting in the loss of statistical significance for the odds of response between the treatment groups at Day 84. These results indicate that addition of ICS and LABA therapy to LAMA monotherapy improves not only lung function but also health status in patients with symptomatic COPD with moderate-to-very-severe airflow limitation. These findings are consistent with previous studies showing that multiple-inhaler triple therapy significantly improved health status, as measured by SGRQ score, versus TIO monotherapy^5–9^. The early decreases in CAT and SGRQ total scores, seen within 28 days in the current study, are notable given that in most previous studies changes in SGRQ score were only assessed after ≥12 weeks of treatment^5–7,9^. Together, these data suggest that single-inhaler FF/UMEC/VI triple therapy leads to relatively rapid improvements in patient symptoms and quality of life in patients with symptomatic COPD with moderate-to-very-severe airflow limitation.

The post hoc subgroup analysis, conducted according to airflow limitation at screening, demonstrated significant improvements in lung function with FF/UMEC/VI versus TIO both in patients with FEV_1_ < 50% predicted and ≥50% predicted at baseline. These findings are consistent with a previous study, which showed that lung function benefits in patients with COPD treated with fluticasone/salmeterol plus TIO versus TIO monotherapy were more pronounced for those with severe airflow limitation (FEV_1_ < 50% predicted)^4^. Additionally, patients with FEV_1_ < 50% predicted at baseline experienced significant decreases in SGRQ total score and CAT score at Days 28 and 84, while those with FEV_1_ ≥ 50% predicted experienced numerical decreases in both scores at each time point that only reached significance at Day 84 for SGRQ total score. These data suggest that a step-up from TIO monotherapy to single-inhaler FF/UMEC/VI triple therapy improves clinical outcomes for patients with symptomatic COPD regardless of airflow limitation, with particular benefit for patients with severe airflow limitation (FEV_1_ < 50% predicted).

Few patients experienced a moderate/severe exacerbation in either treatment group, despite the population being at risk for exacerbation based on the inclusion criterion of FEV_1_ < 50% predicted or <80% predicted with a documented history of ≥2 moderate or 1 severe exacerbation in the 12 months prior to screening. The low overall number of exacerbations is likely due to the short length of the study, which along with the size of the population leaves the study underpowered to detect a between-group difference in the rate of exacerbations. Nonetheless, the proportion of patients experiencing a moderate/severe COPD exacerbation during the study was numerically higher in TIO-treated patients compared with those receiving FF/UMEC/VI. These data are consistent with a real-world observational study that showed a lower risk of COPD exacerbations in patients receiving triple therapy with fluticasone-salmeterol plus TIO compared with TIO alone^15^.

The safety profile of FF/UMEC/VI was similar to that of TIO, with no unexpected safety findings. Rates of SAEs and AESIs, including pneumonia and cardiovascular effects, were low and consistent with previous studies comparing multiple-inhaler triple therapy with TIO monotherapy^4–10^. The low pneumonia rates are reassuring given the association seen between pneumonia and ICS use in previous studies^16^.

Overall, these data show that direct escalation from TIO monotherapy to single-inhaler FF/UMEC/VI triple therapy led to rapid improvements in lung function, symptoms, and health status without an increased risk of pneumonia or other AEs in patients with symptomatic COPD with moderate-to-very-severe airflow limitation. Study limitations include the short study length, which may limit data interpretation. As such, a 1-year study focusing on other outcomes, including the rate of COPD exacerbations, is required. Nonetheless, these data provide valuable clinical information to inform treatment decisions for patients on LAMA monotherapy who continue to experience symptoms and/or exacerbations.

This study demonstrated superiority of once-daily single-inhaler FF/UMEC/VI versus TIO for lung function and patient health status, with a similar safety profile and no difference in pneumonia rates, in patients with symptomatic COPD with moderate-to-very-severe airflow limitation. These results suggest that FF/UMEC/VI is a viable treatment step-up option for optimizing outcomes in patients who continue to experience symptoms and/or exacerbations while receiving LAMA monotherapy.

## Methods

### Trial design

Study 207626 (NCT03474081) was a 12-week, Phase IV, parallel-group, active-controlled, double-blind, double-dummy, randomized, multicenter study comparing once-daily single-inhaler FF/UMEC/VI with TIO monotherapy in patients with symptomatic COPD and moderate-to-very-severe airflow limitation. The study was conducted in 72 centers in three countries (Poland, Russian Federation, and the USA) from March 2018 to July 2019.

Eligible patients were instructed on the proper use of the ELLIPTA and HandiHaler devices at a screening visit (Visit 1) before entering a 4-week run-in period during which they received open-label TIO 18 mcg once daily via HandiHaler and placebo once daily via ELLIPTA. Eligible patients were then randomized 1:1 (using an Interactive Web Response System) to receive either FF/UMEC/VI 100/62.5/25 mcg via ELLIPTA and placebo via HandiHaler or TIO 18 mcg via HandiHaler and placebo via ELLIPTA, all taken once daily in the morning (Visit 2). A double-dummy design was used to ensure blinding, with each patient given two inhalers (ELLIPTA and HandiHaler) to administer the active medication and placebo, and patients self-administered treatment each day. All site personnel involved in efficacy and safety assessments were also blinded to assigned treatment during the study. Rescue albuterol/salbutamol was available as needed throughout the study but withheld for ≥4 hours prior to spirometry assessments. Patients attended two on-treatment study visits (Day 28 [Visit 3] and Day 84 [Visit 4]). Final clinical assessments were conducted on Day 85 (Visit 5). A safety follow-up telephone call or on-site visit (Visit 6) was conducted ~7 days after Visit 5, at the study treatment discontinuation visit, or at the end of the study, whichever was first.

All study patients provided written informed consent. The study was approved by a national, regional, or investigational center ethics committee or institutional review board, in accordance with the International Council on Harmonization of Technical Requirements for Registration of Pharmaceuticals for Human Use Good Clinical Practice and applicable country-specific requirements. Further details are provided in Table 4.

**Table 4:** Institutional Review Board approval numbers by country.

Country	Name, city	Initial approval number	Amendment approval number
Poland	BIOETHICS COMMITTEE at the Regional Medical Chamber in Białystok, Białystok	5/2018/VII	N/A
Russian Federation	Ethics Committee of GBOU VPO Saratov State Medical University named after V.I., Saratov	4063977-20-1	4081983-20-1/IIII
	Best Clinical Practice, Saint Petersburg		
	City Clinical Hospital of Emergency #2, Novosibirsk, Russian Federation		
	Budgetary Healthcare Institution of the Voronezh region “Voronezh Regional Clinical Hospital # 1”, Voronezh		
	Novosibirsk State Regional Clinical Hospital, Novosibirsk		
	Limited Liability Company Medical Association New Hospital, Ekaterinburg		
	City Clinical Hospital #4, Ivanovo		
	Medical Research Institute, St Petersburg		
	Ulyanovsk Regional Clinical Hospital, Ulyanovsk		
	City Clinical Hospital Number 13, Moscow		
	Moscow City Ethical Committee, Moscow		
	Saint-Petersburg SBHI “City Pokrovskaya hospital”, Saint Petersburg		
	GOU VPO Saint Petersburg State Medical University “I.P.Pavlova”, Saint Petersburg		
	FSBI Scientific Research Institute of Pulmonology of FMBA, Moscow		
USA	Western Institutional Review Board, Puyallup, Washington	201800018	MOD00288865
	Advarra Institutional Review Board, Columbia, Maryland		

### Trial population

At screening, eligible patients were ≥40 years of age, current or former smokers with a history of ≥10 pack-years, had an established clinical history of COPD, had been receiving daily COPD maintenance treatment with TIO alone for ≥3 months, had a post-bronchodilator forced expiratory volume in 1 s (FEV_1_) of <50% predicted (or a post-bronchodilator FEV_1_ < 80% predicted and a documented history of ≥2 moderate exacerbations [worsening COPD symptoms requiring treatment with oral/systemic corticosteroids and/or antibiotics] or ≥1 severe exacerbation [worsening COPD symptoms requiring in-patient hospitalization] in the last 12 months), and had a COPD Assessment Test (CAT) score ≥10.

Patients with a current diagnosis of asthma, other respiratory disorders, or other clinically significant diseases were excluded, although participants with a prior history of asthma were eligible if they had a current diagnosis of COPD. Also excluded were those with α1-antitrypsin deficiency as the underlying cause of COPD, a lung resection in the last 12 months, risk factors for pneumonia or recent pneumonia and/or a moderate or severe COPD exacerbation that had not resolved ≥14 days prior to screening and ≥30 days following the last dose of oral/systemic corticosteroids, a respiratory tract infection that had not resolved ≥7 days prior to screening, or an abnormal chest x-ray at or 3 months prior to screening.

Patients were not eligible to be randomized to study treatment if they had a CAT score <10 at Visit 2, demonstrated lack of compliance to run-in treatment (<80% or >120% compliant with either ELLIPTA or HandiHaler), experienced pneumonia, had a moderate or severe COPD exacerbation, or required a change in COPD medication during the run-in period. Assessment of compliance with study treatment between visits was conducted through patient conversations and recording the number of doses left in the ELLIPTA device and the number of capsules dispensed through the HandiHaler. Full inclusion, exclusion, and randomization criteria are provided in Supplementary Note 1.

### Efficacy endpoints

The primary endpoint was change from baseline in trough FEV_1_ at Day 85. To provide a reliable measurement of on-treatment trough FEV_1_ on Day 85, the final dose of study treatment was administered in clinic on Day 84, to ensure high adherence to dosing. Secondary endpoints were change from baseline in trough FEV_1_ at Days 28 and 84. Other endpoints included: change from baseline in SGRQ total score at Days 28 and 84; proportion of SGRQ total score responders at Days 28 and 84 (defined as ≥4-unit decrease in SGRQ total score from baseline); change from baseline in CAT score at Days 28 and 84; proportion of CAT score responders at Days 28 and 84 (defined as ≥2-unit decrease in CAT score from baseline); and moderate or severe exacerbation events. Subgroup analyses by percent predicted FEV_1_ at screening (FEV_1_ < 50% or ≥50%) were performed post hoc.

### Safety assessments

On-treatment AEs were defined as those occurring from the day of starting randomized study treatment until 1 day after stopping randomized study treatment. Incidences of on-treatment adverse events (AEs), including AEs of special interest (AESIs), and serious AEs (SAEs) were recorded. AESIs included cardiovascular effects, decreased bone mineral density and associated fractures, pneumonia, and lower respiratory tract infection (excluding pneumonia). All pneumonias we confirmed clinically and by x-ray, as detailed in Supplementary Note 1.

### Statistical analysis

Sample size was based on the primary endpoint of trough FEV_1_ at Day 85 and assumed 90% power, a two-sided 1% significance level, an estimate of residual standard deviation of 240 mL (based on mixed model repeated measures [MMRM] analyses of the Phase III IMPACT study)^13^ and a treatment difference of 70 mL. Under these assumptions, a total of 702 evaluable patients (351 per treatment group) were required. Assuming an 8% withdrawal rate during the run-in period and 10% withdrawal rate during the study period, it was aimed to enroll ~848 patients into the 4-week run-in period in order to randomize 780 patients.

The intent-to-treat (ITT) population included all randomized patients, excluding those randomized in error, and was used for the analyses of study population, efficacy, and safety. A participant who was recorded as a screen or run-in failure and also randomized but who did not receive any dose of study treatment was considered to be randomized in error. Any participant who received a randomization number was considered to have been randomized.

Both primary and secondary lung function endpoints were analyzed using MMRM, with covariates of baseline FEV_1_, visit, geographical region, and treatment; interaction terms included visit-by-baseline FEV_1_. A visit-by-treatment interaction term was also included to allow treatment effects to be estimated at each visit separately. The variance-covariance matrix was assumed to be unstructured. The primary treatment effect was estimated using a hypothetical strategy that only data up to the time of treatment discontinuation was used in the analysis and data following treatment discontinuation was assumed to follow the same pattern as if the patients had remained on treatment, ie. missing at random.

CAT score and SGRQ total score were analyzed using MMRM, including covariates of baseline value, visit, geographical region, and treatment; interaction terms included visit-by-baseline value and visit-by-treatment. The proportions of CAT or SGRQ responders were analyzed using a generalized linear mixed model with a logit link function and covariates of baseline score, geographical region, treatment group, visit, and visit-by-baseline and visit-by-treatment interactions. TIO was used as the reference level for treatment.

Safety endpoints were analyzed in the ITT population using descriptive statistics. AESIs were defined as AEs that have specified areas of interest for FF, UMEC, and VI, or the overall COPD population.

### Reporting summary

Further information on research design is available in the Nature Research Reporting Summary linked to this article.

## Supplementary information

Reporting Summary

Supplementary Information

## Data Availability

Anonymized individual participant data and study documents can be requested for further research from www.clinicalstudydatarequest.com by submitting an enquiry citing GSK study number 207626.
