# Supplementary material for: Single-inhaler fluticasone furoate/umeclidinium/vilanterol (FF/UMEC/VI) triple therapy versus tiotropium monotherapy in patients with COPD
Source: NPJ Prim Care Respir Med. 2021 May 25;31:29. doi: 10.1038/s41533-021-00241-z (PMC8149706; doi:10.1038/s41533-021-00241-z)
Supplement: Supplementary file 1 — Reporting Summary [file 41533_2021_241_MOESM1_ESM.pdf]

nature research

Corresponding author(s): NPJPCRM-01173
Last updated by author(s): Feb 25, 2021

Reporting Summary

Nature Research wishes to improve the reproducibility of the work that we publish. This form provides structure for consistency and transparency in reporting. For further information on Nature Research policies, see our Editorial Policies and the Editorial Policy Checklist.

Statistics

For all statistical analyses, confirm that the following items are present in the figure legend, table legend, main text, or Methods section.

n/a	Confirmed	
☐	☒	The exact sample size (*n*) for each experimental group/condition, given as a discrete number and unit of measurement
☒	☐	A statement on whether measurements were taken from distinct samples or whether the same sample was measured repeatedly
☐	☐	The statistical test(s) used AND whether they are one- or two-sided<br>*Only common tests should be described solely by name; describe more complex techniques in the Methods section.*
☐	☒	A description of all covariates tested
☐	☒	A description of any assumptions or corrections, such as tests of normality and adjustment for multiple comparisons
☐	☒	A full description of the statistical parameters including central tendency (e.g. means) or other basic estimates (e.g. regression coefficient) AND variation (e.g. standard deviation) or associated estimates of uncertainty (e.g. confidence intervals)
☐	☒	For null hypothesis testing, the test statistic (e.g. *F*, *t*, *r*) with confidence intervals, effect sizes, degrees of freedom and *P* value noted<br>*Give P values as exact values whenever suitable.*
☒	☐	For Bayesian analysis, information on the choice of priors and Markov chain Monte Carlo settings
☒	☐	For hierarchical and complex designs, identification of the appropriate level for tests and full reporting of outcomes
☒	☐	Estimates of effect sizes (e.g. Cohen's *d*, Pearson's *r*), indicating how they were calculated

Our web collection on statistics for biologists contains articles on many of the points above.

Software and code

Policy information about availability of computer code

Data collection	*Provide a description of all commercial, open source and custom code used to collect the data in this study, specifying the version used OR state that no software was used.*
Data analysis	*Provide a description of all commercial, open source and custom code used to analyse the data in this study, specifying the version used OR state that no software was used.*

For manuscripts utilizing custom algorithms or software that are central to the research but not yet described in published literature, software must be made available to editors and reviewers. We strongly encourage code deposition in a community repository (e.g. GitHub). See the Nature Research guidelines for submitting code & software for further information.

Data

Policy information about availability of data

All manuscripts must include a data availability statement. This statement should provide the following information, where applicable:

- Accession codes, unique identifiers, or web links for publicly available datasets
- A list of figures that have associated raw data
- A description of any restrictions on data availability

Anonymized individual participant data and study documents can be requested for further research from www.clinicalstudydatarequest.com.

Field-specific reporting

Please select the one below that is the best fit for your research. If you are not sure, read the appropriate sections before making your selection.

☒ Life sciences ☐ Behavioural & social sciences ☐ Ecological, evolutionary & environmental sciences

For a reference copy of the document with all sections, see nature.com/documents/nr-reporting-summary-flat.pdf

Life sciences study design

All studies must disclose on these points even when the disclosure is negative.

Sample size	Sample size was based on the primary endpoint of trough FEV1 at Day 85 and assumed 90% power, a two-sided 1% significance level, an estimate of residual standard deviation of 240 mL (based on mixed model repeated measures [MMRM] analyses of the Phase III IMPACT study)13 and a treatment difference of 70 mL. Under these assumptions, a total of 702 evaluable patients (351 per treatment group) were required. Assuming an 8% withdrawal rate during the run-in period and 10% withdrawal rate during the study period, it was aimed to enroll approximately 848 patients into the 4-week run-in period in order to randomize 780 patients.
Data exclusions	The intent-to-treat (ITT) population included all randomized patients, excluding those randomized in error, and was used for the analyses of study population, efficacy, and safety. A participant who was recorded as a screen or run-in failure and also randomized but who did not receive any dose of study treatment was considered to be randomized in error. Any other participant who received a randomization number was considered to have been randomized.
Replication	N/A
Randomization	Participants were assigned to study treatment in accordance with the randomization schedule. The randomization code was generated using a validated computerized system. Participants were randomized using an Interactive Web Response System (IWRS). The study used central-based randomization to allocate treatments. Once a randomization number had been assigned to a participant it could not be reassigned to any other participant in the study.<br><br>Participants were randomly assigned in a 1:1 ratio to receive either:<br>• FF/UMEC/VI 100/62.5/25 mcg via Ellipta once-daily in the morning + placebo to match TIO via HandiHaler once-daily in the morning<br>• TIO 18 mcg via HandiHaler once-daily in the morning* + placebo to match FF/UMEC/VI via Ellipta once-daily in the morning.
Blinding	This was a double-blind, double-dummy study. Participants were given 2 inhalers (Ellipta and HandiHaler), 1 of which contained placebo based on their randomization assignment. All participants and site personnel involved in efficacy and safety assessments were blinded to assigned treatment during the study. Tiotropium capsules had trade markings that were not present on the placebo capsules. The parallel-group design of the study ensured the capsule type was consistent for each participant for the duration of the study. In addition, TIO and placebo capsules were closely matched in color. Both the TIO and placebo blister packages were covered with opaque over-labels with the intent of hiding the information on the TIO packaging. The HandiHaler devices were covered with labels to mask identifying marks on the inhaler. Investigators and site personnel involved in efficacy and safety assessments were instructed to not be present when a participant administered their study medication at study visits, to guard against the possibility of personnel identifying the capsules removed from the blisters. Sites were required to have study treatment dosed, dispensed and accounted for by site personnel that were not involved in any efficacy or safety assessments. Treatment codes could be unblinded by the investigator or treating physician only in the case of a medical emergency or in the event of a serious medical condition, when knowledge of the IP was essential for the clinical management or welfare of the participant. GSK Global Clinical Safety and Pharmacovigilance (GCSP) staff could unblind treatment codes in the event of an SAE.

Reporting for specific materials, systems and methods

We require information from authors about some types of materials, experimental systems and methods used in many studies. Here, indicate whether each material, system or method listed is relevant to your study. If you are not sure if a list item applies to your research, read the appropriate section before selecting a response.

Materials & experimental systems

n/a	Involved in the study	
☒	☐	Antibodies
☒	☐	Eukaryotic cell lines
☒	☐	Palaeontology and archaeology
☒	☐	Animals and other organisms
☐	☒	Human research participants
☐	☒	Clinical data
☒	☐	Dual use research of concern

Methods

n/a	Involved in the study	
☒	☐	ChIP-seq
☒	☐	Flow cytometry
☒	☐	MRI-based neuroimaging

Human research participants

Policy information about studies involving human research participants

Population characteristics	This study was designed to evaluate the impact of switching participants from LAMA monotherapy (TIO) to single inhaler triple therapy (FF/UMEC/VI) on lung function and health status. The majority of participants enrolled had moderate to severe COPD (92%), 8% had very severe COPD, and the majority of participants were not reversible to albuterol/salbutamol at Screening (82%). Study participants had significant airflow limitation as characterized by a low percent predicted post-bronchodilator FEV1 (50.0%) at Screening, and poor health status as indicated by high SGRQ Total Scores at baseline and high CAT scores at Screening and baseline. A high proportion of participants (53%) had experienced at least 2 moderate exacerbations in the last 12 months, and 22% had experienced at least 1 severe exacerbation. Therefore, based on airflow obstruction and/or an exacerbation history, the study population represented a severe and symptomatic COPD population at risk of exacerbation despite maintenance therapy. Baseline demographic characteristics were similar between the treatment groups. The study enrolled a larger proportion of males (68%) than females (32%), and 52% of participants were former smokers. Half of the study participants were recruited in Poland, 25% in the USA, and 25% in the Russian Federation. A low proportion of participants discontinued study treatment (3%) and/or withdrew from study (4%). During the 4-week Run-in Period, all participants received TIO 18 mcg as Run-in treatment; COPD maintenance treatment with daily TIO alone for at least 3 months prior to Screening was an inclusion criterion for this study.
Recruitment	*Describe how participants were recruited. Outline any potential self-selection bias or other biases that may be present and how these are likely to impact results.*
Ethics oversight	The study was approved by a national, regional, or investigational center ethics committee or institutional review board, in accordance with the International Council on Harmonization of Technical Requirements for Registration of Pharmaceuticals for Human Use Good Clinical Practice and applicable country-specific requirements as follows:<br>Poland - Initial approval number: 5/2018/VII N<br>Russian Federation - Initial approval number: 4063977-20-1 Amendment approval number: 4081983-20-1/IIII<br>USA - Initial approval number: 201800018 V: MOD00288865

Note that full information on the approval of the study protocol must also be provided in the manuscript.

Clinical data

Policy information about clinical studies

All manuscripts should comply with the ICMJE guidelines for publication of clinical research and a completed CONSORT checklist must be included with all submissions.

Clinical trial registration	GSK study 207636; NCT03474081
Study protocol	https://clinicaltrials.gov/ct2/show/NCT03474081
Data collection	Participant data was entered into PAREXEL-defined eCRFs, transmitted electronically to GSK and combined with data provided from other sources (e.g., laboratory data) in a validated data system.<br><br>Participants were consented to participate in the study at the Pre-screening Visit (Visit 0). Eligible participants attended a Screening Visit (Visit 1) during which they were instructed on the proper use of the Ellipta and HandiHaler. Eligible participants entered a 4-week Run-in Period, during which they received open-label TIO 18 mcg once daily via the HandiHaler and placebo via the Ellipta. Participants were provided with rescue albuterol/salbutamol to be used on an as needed basis throughout the study.<br>At the Randomization Visit (Visit 2), participants who met all of the randomization criteria (Section 4.4.1.1) were randomly assigned in a 1:1 ratio to receive eitherFF/UMEC/VI 100/62.5/25 mcg via the Ellipta once daily or TIO 18 mcg via the HandiHaler once daily, and matching placebo. Participants received their randomized study treatment for 84 days, during which they attended 2 on-treatment study Visits (Visit 3 at Week 4 and Visit 4 at Week 12). Final clinical assessments were conducted on Day 85 (Visit 5). A Safety Follow-up telephone contact or on-site visit (Visit 6) was conducted approximately 7 days after the completion of all protocol-defined procedures at Visit 5/End of Study or the Study Treatment Discontinuation Visit.
Outcomes	The primary efficacy endpoint was change from baseline in trough FEV1 at Week 12 (Day 85).<br>The secondary efficacy endpoint was change from baseline in trough FEV1 at Week 4 (Day 28) and Week 12 (Day 84).<br>The other efficacy endpoints were:<br>The proportion of responders based on the St George's Respiratory Questionnaire (SGRQ) Total Score at Week 4 and Week 12;<br>The change from baseline in SGRQ Total Score at Week 4 and Week 12;<br>The proportion of responders based on the CAT Total Score at Week 4 and Week 12;<br>The change from baseline in CAT Total Score at Week 4 and Week 12;<br>A moderate or severe exacerbation event.
