# Supplementary material for: Single-inhaler fluticasone furoate/umeclidinium/vilanterol (FF/UMEC/VI) triple therapy versus tiotropium monotherapy in patients with COPD
Source: NPJ Prim Care Respir Med. 2021 May 25;31:29. doi: 10.1038/s41533-021-00241-z (PMC8149706; doi:10.1038/s41533-021-00241-z)
Supplement: Supplementary file 2 — Supplementary Information [file 41533_2021_241_MOESM2_ESM.pdf]

Supplementary Information for:

Single-inhaler fluticasone furoate/umeclidinium/vilanterol (FF/UMEC/VI) triple therapy versus tiotropium monotherapy: lung function and health status in a randomized controlled trial of patients with COPD

Authors: Sandeep Bansal MD, Martin Anderson MD, PhD, Antonio Anzueto MD, Nicola Brown PhD, Chris Compton MD, Thomas C. Corbridge MD, David Erb MD, Catherine Harvey DPhil, Morrys C. Kaisermann MD, PhD, Mitchell Kaye MD, David A. Lipson MD, Neil Martin MD, Chang-Qing Zhu PhD, Alberto Papi MD

Supplementary Figures

Supplementary Figure 1: ***SGRQ total score percent predicted FEV_1 at screening subgroups (ITT population)***

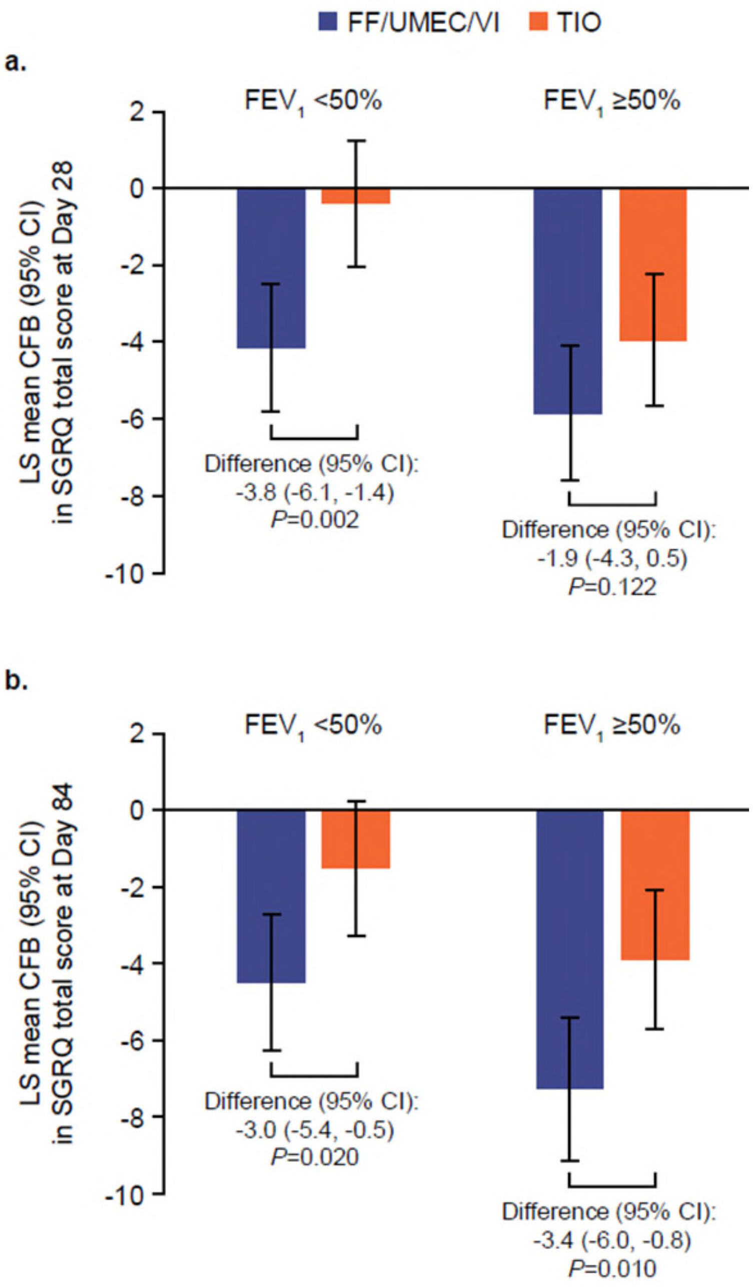

Least squares mean (95% CI) change from baseline in SGRQ total score at (a) Day 28, and (b) Day 84

CFB, change from baseline; CI, confidence interval; FEV_1, forced expiratory volume in 1 second; FF, fluticasone furoate; ITT, intent-to-treat; LS, least squares; TIO, tiotropium, UMEC, umeclidinium; VI, vilanterol.

Supplementary Figure 2: ***CAT Score for percent predicted FEV_1 at screening subgroups (ITT population)***

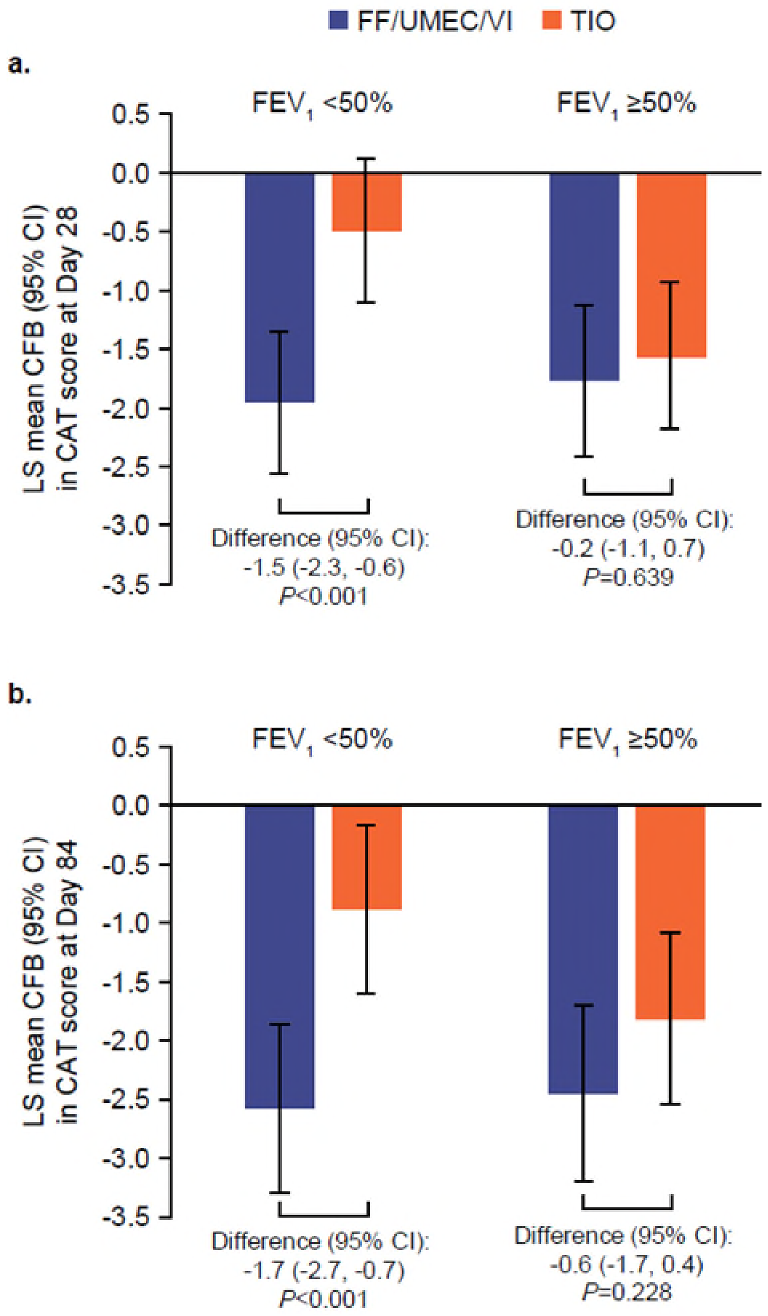

Least squares mean (95% CI) change from baseline in CAT score at (a) Day 28 and (b) Day 84

CFB, change from baseline; CI, confidence interval; FEV_1, forced expiratory volume in 1 second; FF, fluticasone furoate; ITT, intent-to-treat; LS, least squares; TIO, tiotropium, UMEC, umeclidinium; VI, vilanterol.

Supplementary Notes

Supplentary Note 1: *Additional methods*

Inclusion Criteria

Participants were eligible to be included in the study only if all of the following criteria applied:

- Informed consent: capable of giving signed informed consent prior to study start, which included compliance with the requirements and restrictions listed in the informed consent form and in this protocol.
- Type of participant: Outpatient.
- Age: Participants were 40 years of age or older at screening (Visit 1).
- Gender: Male or female participants.

  Female participants: A female participant was eligible to participate if she was not pregnant, not breastfeeding, and at least one of the following conditions applied:

  a. Not a woman of childbearing potential (WOCBP)

  OR

  b. A WOCBP who agreed to follow the contraceptive guidance in Appendix 3 during the treatment period and until the safety follow-up contact after the last dose of study treatment.
- COPD diagnosis: An established clinical history of COPD in accordance with the definition by the American Thoracic Society/European Respiratory Society.[1]
- Smoking history: Current or former cigarette smokers with a history of cigarette smoking of ≥10 pack-years at screening (Visit 1) (number of pack years = [number of cigarettes per day/20] x number of years smoked [eg, 20 cigarettes per day for 10 years, or 10 cigarettes per day for 20 years]). Previous smokers were defined as those who had stopped smoking for at least 6 months prior to Visit 1. Note: Pipe and/or cigar use could not be used to calculate pack-year history.

- Severity of COPD symptoms: A score of ≥10 on the COPD Assessment Test (CAT) at screening (Visit 1).
- Severity of Disease: Participants were required to demonstrate at screening: a post-bronchodilator forced expiratory volume in 1 second (FEV_1) <50% predicted normal OR a post-bronchodilator FEV_1 <80% predicted normal and a documented history of ≥2 moderate exacerbations or one severe (hospitalized) exacerbation in the previous 12 months. Participants were also required to have a measured post-albuterol/salbutamol FEV_1/forced vital capacity (FVC) ratio of <0.70 at screening. Note: Percent predicted was calculated using the European Respiratory Society Global Lung Function Initiative reference equations[2]; Note: A documented history of a COPD exacerbation (eg, medical record verification) was a medical record of worsening COPD symptoms that required systemic/oral corticosteroids and/or antibiotics (for a moderate exacerbation) or hospitalization (for a severe exacerbation). Prior use of antibiotics alone did not qualify as an exacerbation history unless the use was associated with treatment of worsening symptoms of COPD, such as increased dyspnea, sputum volume, or sputum purulence (color). Participant verbal reports were not acceptable.
- Existing COPD maintenance treatment: participant must have been receiving daily maintenance treatment with tiotropium alone (via the HandiHaler or Respimat) for their COPD for at least 3 months prior to screening. (Note: Participants taking only as-needed tiotropium were not eligible).

Exclusion Criteria

Participants were excluded from the study if any of the following criteria applied:

- Pregnancy: Women who were pregnant or lactating or were planning on becoming pregnant during the study.

- Asthma: Participants with a current diagnosis of asthma (participants with a prior history of asthma were eligible if they had a current diagnosis of COPD).
- α1-antitrypsin deficiency: Participants with α1-antitrypsin deficiency as the underlying cause of COPD.
- Other respiratory disorders: Participants with active tuberculosis, lung cancer, and clinically significant bronchiectasis, sarcoidosis, lung fibrosis, pulmonary hypertension, interstitial lung disease, or other active pulmonary diseases.
- Lung resection: Participants who had undergone lung volume reduction surgery within the 12 months prior to screening.
- Risk factors for pneumonia: Immune suppression (eg, advanced human immunodeficiency virus with high viral load and low CD4 count, lupus on immunosuppressants) that in the opinion of the investigator would increase risk of pneumonia or other risk factors for pneumonia (eg, neurological disorders affecting control of the upper airway, such as Parkinson's disease, myasthenia gravis). Participants at potentially high risk for pneumonia (eg, very low body mass index, severely malnourished, or very low FEV_1) were only included at the discretion of the Investigator.
- Pneumonia and/or moderate or severe COPD exacerbation that had not resolved at least 14 days prior to screening and at least 30 days following the last dose of oral/systemic corticosteroids (if applicable).
- Respiratory tract infection that had not resolved at least 7 days prior to screening.
- Abnormal chest x-ray: Chest x-ray (posteroanterior and lateral) revealing evidence of pneumonia or a clinically significant abnormality not believed to be due to the presence of COPD, or another condition that would hinder the ability to detect an infiltrate on chest x-ray (eg, significant cardiomegaly, pleural effusion, or scarring). All participants had a chest x-ray at Screening Visit 1 (or historical radiograph or computerized tomography [CT] scan obtained within 3 months prior to screening). Note: Participants who had experienced

pneumonia and/or moderate or severe COPD exacerbations within 3 months of screening were required to provide a post pneumonia/exacerbation chest x-ray or have a chest x-ray conducted at screening. For sites in Germany: If a chest x-ray (or CT scan) within 3 months prior to screening (Visit 1) was not available, approval to conduct a diagnostic chest x-ray had to be obtained from the Federal Office for Radiation Protection (BfS).

- Other diseases/abnormalities: Participants with historical or current evidence of clinically significant cardiovascular, neurological, psychiatric, renal, hepatic, immunological, gastrointestinal, urogenital, nervous system, musculoskeletal, skin, sensory, endocrine (including uncontrolled diabetes or thyroid disease), or hematological abnormalities that were uncontrolled. Significant was defined as any disease that, in the opinion of the Investigator, would put the safety of the participant at risk through participation, or which would affect the efficacy or safety analysis if the disease/condition exacerbated during the study.
- Unstable liver disease: ALT >2x upper limit of normal (ULN); and bilirubin >1.5x ULN (isolated bilirubin >1.5x ULN was acceptable if bilirubin was fractionated and direct bilirubin <35%). Current active liver or biliary disease (with the exception of Gilbert's syndrome or asymptomatic gallstones or otherwise stable chronic liver disease per investigator assessment). Notes: Stable chronic liver disease was generally defined by the absence of ascites, encephalopathy, coagulopathy, hypoalbuminemia, esophageal or gastric varices, or persistent jaundice, or cirrhosis. Chronic stable hepatitis B and C (eg, presence of hepatitis B surface antigen or positive hepatitis C antibody test result at screening or within 3 months prior to first dose of study treatment) were acceptable if participant otherwise met entry criteria.
- Unstable or life-threatening cardiac disease: Participants with any of the following at screening (Visit 1) were excluded:

  a. Myocardial infarction or unstable angina in the last 6 months

b. Unstable or life-threatening cardiac arrhythmia requiring intervention in the last 3 months

c. New York Heart Association Class IV heart failure.

- Abnormal and clinically significant 12-lead ECG finding at Visit 1. The Investigator determined the clinical significance of each abnormal ECG finding in relation to the participant's medical history and excluded participants who would be at undue risk by participating in the trial. An abnormal and clinically significant finding that would preclude a participant from entering the trial was defined as a 12-lead ECG tracing that was interpreted at, but not limited to, any of the following:

  a. Atrial fibrillation with rapid ventricular rate >120 beats per minute

  b. Sustained and non-sustained ventricular tachycardia

  c. Second-degree heart block Mobitz type II and third degree heart block (unless pacemaker or defibrillator had been inserted)

  d. QT interval corrected for heart rate ≥500 msec in participants with QRS <120 msec and QTcF ≥530 msec in participants with QRS ≥120 msec.

- Contraindications: A history of allergy or hypersensitivity to any corticosteroid, anticholinergic/muscarinic receptor antagonist, β_2-agonist, lactose/milk protein or magnesium stearate or a medical condition such as narrow-angle glaucoma, prostatic hypertrophy or bladder neck obstruction that, in the opinion of the Investigator, contraindicated study participation.
- Cancer: Participants with carcinoma that had not been in complete remission for at least 3 years. Participants who had carcinoma in situ of the cervix, squamous cell carcinoma, and basal cell carcinoma of the skin would not be excluded based on the 3-year waiting period if the participant had been considered cured by treatment.
- Oxygen therapy: Use of long-term oxygen therapy described as resting oxygen therapy >3 L/min (oxygen use ≤3 L/min flow is not exclusionary).

- Medication prior to spirometry: Participants who were medically unable to withhold their albuterol/salbutamol for the 4-hour period required prior to spirometry testing at each study visit.
- Pulmonary rehabilitation: Participants who had participated in the acute phase of a pulmonary rehabilitation program within 4 weeks prior to screening or participants who planned to enter the acute phase of a pulmonary rehabilitation program during the study. Participants who were in the maintenance phase of a pulmonary rehabilitation program were not excluded.
- Drug/alcohol abuse: Participants with a known or suspected history of alcohol or drug abuse within the last 2 years.
- Non-compliance: Participants who were at risk of non-compliance, or unable to comply with the study procedures. Any infirmity, disability, or geographic location that would limit compliance for scheduled visits.
- Questionable validity of consent: Participants with a history of psychiatric disease, intellectual deficiency, poor motivation, or other conditions that would limit the validity of informed consent to participate in the study.
- Affiliation with Investigator site: Study Investigators, sub-Investigators, study coordinators, employees of a participating Investigator or study site, or immediate family members of the aforementioned that were involved with this study.
- Inability to read: In the opinion of the Investigator, any participant who was unable to read and/or would not have been able to complete study related materials.
- Medication prior to screening: Use of the following medications within the following time intervals prior to Visit 1.

Medication	No use within the following time intervals prior to screening
Inhaled short-acting anticholinergics	6 hours
Inhaled short-acting β_2-agonists*	≥4 hours
Inhaled short-acting anticholinergics + short-acting β_2-agonist combination	6 hours
Inhaled corticosteroids (ICS)	Use as maintenance treatment in the 3 months prior to Visit 1 was not permitted.<br><br>Maintenance treatment was defined as use for ≥14 consecutive days (at any time in the 3 months prior to Visit 1).
ICS/inhaled long-acting β_2-agonist (LABA) combinations (eg, fluticasone/salmeterol, mometasone furoate/formoterol fumarate, budesonide/formoterol fumarate, fluticasone furoate/vilanterol)	
Phosphodiesterase 4 (PDE4) inhibitors (roflumilast)	
LABA (eg, indacaterol, olodaterol)	
Long acting muscarinic antagonists[†] (LAMA) (eg, umeclidinium, aclidinium, glycopyrronium)	
LAMA/LABA combinations	
Theophyllines	
Sodium cromoglycate and nedocromil sodium	
Anti-leukotrienes	
Long-term antibiotic therapy	Participants receiving antibiotics for long-term therapy were not eligible for the study. (Antibiotics were allowed for the short-term treatment [≤14 days] of an exacerbation or for short-term treatment [≤14 days] of other acute infections during the study).
Systemic, oral, parenteral corticosteroids	30 days<br>(During the study oral/systemic corticosteroids could be used for ≤14 days to treat COPD exacerbations/pneumonia). Intra-articular injections were allowed.
Any other investigational drug	30 days or 5 half-lives, whichever is longer.

*Rescue albuterol/salbutamol was provided and was permitted during the study.

[†]Excluding tiotropium, which was required to have been taken for at least 3 months prior to screening.

Randomization Criteria

At the end of the run-in period (Visit 2), study participants were required to fulfil the following additional criteria in order to be randomized into the study and enter the treatment period:

- CAT score at randomization (Visit 2): Participants with CAT score <10 at randomization (Visit 2) were excluded.
- Compliance with run-in study medication: Compliance with each run-in study medication was assessed by the Investigator, and any participant <80% or >120% compliant with either of the two inhalers (ELLIPTA or HandiHaler) was excluded.
- COPD exacerbation or pneumonia: Participants that experienced a moderate or a severe COPD exacerbation or pneumonia during the run-in period were excluded.
- Changes in COPD medication: Any participant that required a change in COPD medication from tiotropium (18 mcg) and rescue medication (albuterol/salbutamol) during the run-in period was excluded. This included a temporary change in COPD medication.

Diagnosis of pneumonia

All suspected pneumonias required confirmation as defined by the presence of new infiltrate(s) on chest x-ray and at least two of the following signs and symptoms: increased cough; increased sputum purulence or production; auscultatory findings of adventitious sounds; dyspnea or tachypnea; fever (oral temperature >37.5^{o}C); elevated white blood cells (>10,000/mm^3 or >15% immature forms); hypoxemia (HbO_2 saturation <88% or at least 2% lower than baseline value).

References

1. Celli B. R., MacNee W. & ATS/ERS Task Force. Standards for the diagnosis and treatment of patients with COPD: A summary of the ATS/ERS position paper. *Eur. Respir. J.* **23**, 932–946 (2004).
2. Quanjer P. *et al.* Multi-ethnic reference values for spirometry for the 3-95-yr age range: The global lung function 2012 equations. *Eur. Respir. J.* **40**, 1324–1343 (2012).
